# Supplementary material for: Machine Learning Improves the Prediction Rate of Non-Curative Resection of Endoscopic Submucosal Dissection in Patients with Early Gastric Cancer
Source: Cancers (Basel). 2022 Jul 31;14(15):3742. doi: 10.3390/cancers14153742 (PMC9367410; doi:10.3390/cancers14153742)
Supplement: Supplementary file 1 [file cancers-14-03742-s001.zip › cancers-1810287-supplementary.pdf]

cancers

MDPI

Supplementary Materials

Machine Learning Improves the Prediction Rate of Non-Curative Resection of Endoscopic Submucosal Dissection in Patients with Early Gastric Cancer

Hae-Ryong Yun, Cheal Wung Huh, Da Hyun Jung, Gyubok Lee, Nak-Hoon Son, Jie-Hyun Kim, Young Hoon Youn, Jun Chul Park, Sung Kwan Shin, Sang Kil Lee, Yong Chan Lee

Supplementary Table S1. Baseline characteristics of the non-curative and curative resection groups

Supplementary Table S2. Performance of the machine learning model according to different threshold values

Supplementary Table S3. Performance of the risk-scoring system in predicting non-curative resection

Supplementary Table S4. Performance of the non-curative resection prediction model between the seven machine learning models

Supplementary Figure S1. Variables ordered by XGBoost feature importance and domain expertise Abbreviations: AMD, adenocarcinoma moderate-differentiated; AWD, adenocarcinoma well-differentiated; SRC, signet-ring cell; APD, adenocarcinoma poorly-differentiated; CIS, carcinoma in situ; SCC, squamous cell carcinoma.

Supplementary Figure S2. The precision-recall curves for prediction of non-curative resection after endoscopic submucosal dissection (A) Internal data set, (B) External data set. Abbreviation: RSS, risk-scoring system; LR, logistic regression; SVM, support vector machine; KNN, k-nearest neighbors; NB, naive bayes; XGB, extreme gradient boosting; RF, random forest; MLP, multilayer perceptron.

Supplementary Figure S3. Decision curve analysis of extreme gradient boosting and other models. (A) Internal data set, (B) External data set. Abbreviation: LR, logistic regression; SVM, support vector machine; KNN, k-nearest neighbors; NB, naive bayes; XGB, extreme gradient boosting; RF, random forest; MLP, multilayer perceptron.

Supplementary Table S1. Baseline characteristics of the non-curative and curative resection groups

	Internal data set			External data set		
	CR (n=3330)	NCR (n=1066)	*P*-value	CR (n=497)	NCR (n=34)	*P*-value
**Demographics**						
Age, years	65.0 (9.5)	63.9 (11.6)	0.002	62.3 (11.0)	63.2 (1.2)	0.63
Male	2507 (75.3)	733 (68.8)	<0.001	353 (71.0)	27 (79.4)	0.29
**Medications**						
Anti-thrombotics	633 (19.0)	197 (18.5)	0.70	86 (17.3)	7 (20.6)	0.63
**Histology**						
AWD	1264 (38.0)	161 (15.1)	<0.001	136 (27.4)	4 (11.8)	0.04
AMD	751 (22.6)	358 (33.6)	<0.001	107 (21.5)	12 (35.3)	0.06
APD	55 (1.7)	58 (5.4)	<0.001	27 (5.4)	5 (14.7)	0.02
SRC	31 (0.9)	179 (16.8)	<0.001	38 (7.6)	2 (5.9)	0.71
Other (Mucinous, CIS, SCC)	1229 (36.9)	310 (41.7)	<0.001	189 (40.2)	11 (32.3)	0.08
**Multiple lesions**						
1	2857 (85.8)	928 (87.1)	0.30	463 (93.2)	32 (94.1)	0.83
2	417 (12.5)	115 (10.8)	0.13	27 (5.4)	2 (5.9)	0.91
>2	473 (14.2)	138 (12.9)	0.30	34 (6.8)	2 (5.9)	0.83
**Tumor location (long axis)**						
Upper	285 (8.6)	156 (14.6)	<0.001	49 (9.9)	5 (14.7)	0.37
Mid	1038 (31.2)	425 (39.9)	<0.001	201 (40.4)	17 (50.0)	0.27
Lower	2021 (60.7)	348 (32.6)	<0.001	254 (51.1)	12 (35.3)	0.07
**Tumor location (short axis)**						
AW	731 (22.0)	196 (18.4)	0.013	115 (23.1)	8 (23.5)	0.96
PW	775 (23.3)	295 (27.7)	0.004	110 (22.1)	9 (26.5)	0.56

LC	1308 (39.3)	299 (28.0)	<0.001	214 (43.1)	17 (50)	0.43
GC	705 (21.2)	199 (18.7)	0.07	112 (22.5)	7 (20.6)	0.79
**Tumor size (mm)**	11.8 (7.8)	14.1 (9.6)	<0.001	19.6 (12.4)	25.0 (15.1)	0.01
**Endoscopic appearance**						
Elevated	2390 (71.8)	586 (55.0)	<0.001	257 (51.7)	20 (58.8)	0.42
Flat	756 (22.7)	368 (34.5)	<0.001	203 (40.8)	14 (41.2)	0.97
Depressed	1563 (46.9)	466 (43.7)	0.06	254 (51.1)	19 (55.9)	0.59
**Endoscopic finding**						
Ulcer	150 (4.5)	75 (7.0)	0.001	43 (8.7)	6 (17.6)	0.08
Fusion of fold, interruption, or smooth tapering of fold	39 (1.2)	31 (2.9)	<0.001	30 (6.0)	4 (11.8)	0.19
Erythema	426 (12.8)	108 (10.1)	0.021	249 (50.1)	12 (35.3)	0.09
Exudate	59 (1.8)	44 (4.1)	<0.001	103 (20.7)	4 (11.8)	0.21
Whitish scar or atrophy	163 (4.9)	62 (5.8)	0.23	42 (8.5)	2 (5.9)	0.60
Nodularity or elevated	397 (11.9)	199 (18.7)	<0.001	249 (50.1)	18 (52.9)	0.75
Spontaneous bleeding	18 (0.5)	20 (1.9)	<0.001	20 (4.0)	2 (5.9)	0.60

Note: Values for categorical variables are given as a number (percentage); values for continuous variables, as mean (standard deviation).
Abbreviations: AMD, adenocarcinoma moderate-differentiated; AWD, adenocarcinoma well-differentiated; APD, adenocarcinoma poorly-differentiated; SRC, signet-ring cell; CIS, carcinoma in situ; SCC, squamous cell carcinoma; AW, anterior wall; PW, posterior wall; LC, lesser curvature; GC, greater curvature.

Supplementary Table S2. Performance of the machine learning model according to different threshold values

Threshold	Model	Internal data set				External data set			
		Sensitivity	Specificity	Precision	F1 score	Sensitivity	Specificity	Precision	F1 score
0.1	LR	0.481	0.915	0.360	0.517	0.277	0.941	0.081	0.150
	SVM	0.107	0.986	0.261	0.413	0.052	1.000	0.067	0.126
	KNN	0.426	0.918	0.339	0.495	0.255	0.794	0.068	0.125
	NB	0.946	0.380	0.696	0.492	0.746	0.411	0.100	0.160
	XGB	0.531	0.928	0.383	0.542	0.293	0.882	0.078	0.144
	RF	0.050	0.993	0.250	0.400	0.004	1.000	0.064	0.120

	MLP	0.013	1.000	0.245	0.393	0.257	0.911	0.077	0.142
0.3	LR	0.873	0.602	0.603	0.602	0.643	0.676	0.115	0.196
	SVM	0.906	0.491	0.628	0.551	0.698	0.705	0.137	0.230
	KNN	0.877	0.525	0.579	0.550	0.889	0.441	0.202	0.271
	NB	0.946	0.379	0.695	0.491	0.780	0.352	0.099	0.154
	XGB	0.864	0.610	0.590	0.600	0.722	0.617	0.132	0.217
	RF	0.933	0.427	0.671	0.522	0.730	0.558	0.124	0.203
	MLP	0.936	0.441	0.691	0.539	0.643	0.647	0.110	0.188
0.5	LR	0.949	0.436	0.734	0.547	0.770	0.470	0.123	0.195
	SVM	0.951	0.320	0.679	0.435	0.827	0.411	0.140	0.209
	KNN	0.969	0.334	0.777	0.467	0.967	0.117	0.200	0.148
	NB	0.947	0.379	0.690	0.491	0.792	0.323	0.113	0.167
	XGB	0.950	0.468	0.750	0.576	0.843	0.529	0.187	0.276
	RF	0.994	0.197	0.925	0.326	0.935	0.029	0.030	0.029
	MLP	0.995	0.218	0.943	0.354	0.818	0.382	0.126	0.189
0.6	LR	0.982	0.332	0.855	0.478	0.853	0.323	0.131	0.186
	SVM	0.972	0.224	0.722	0.342	0.895	0.147	0.087	0.109
	KNN	0.995	0.245	0.942	0.389	0.985	0.058	0.222	0.093
	NB	0.947	0.379	0.697	0.491	0.827	0.323	0.113	0.167
	XGB	0.977	0.349	0.830	0.492	0.905	0.205	0.129	0.159
	RF	0.999	0.017	0.950	0.035	0.996	0.000	0.000	0.000
	MLP	0.999	0.023	0.961	0.045	0.909	0.147	0.100	0.119
0.9	LR	0.997	0.176	0.954	0.297	0.935	0.058	0.058	0.058
	SVM	0.994	0.094	0.841	0.170	0.975	0.000	0.000	0.000
	KNN	0.998	0.196	0.976	0.326	0.994	0.058	0.400	0.102
	NB	0.948	0.379	0.700	0.492	0.885	0.294	0.149	0.198
	XGB	0.997	0.193	0.967	0.322	0.961	0.058	0.095	0.072
	RF	1.000	0.000	0.000	0.000	1.000	0.000	0.000	0.000
	MLP	1.000	0.000	0.000	0.000	0.983	0.094	0.111	0.046

Abbreviation: LR, logistic regression; SVM, support vector machine; KNN, k-nerest neighbors; NB, naive bayes; XGB, extreme gradient boosting; RF, random forest; MLP, multilayer perceptron.

Supplementary Table S3. Performance of the risk-scoring system in predicting non-curative resection

Risk score	NCR ratio[a]	Sensitivity	Specificity	Precision	F1 score	AUROC (95%CI)
**Internal data**						
0	75/313 (22.9%)	0.0	1.0	0.239	0.386	0.701 (0.683–0.720)
≥1	269/2426 (11.0%)	0.071	0.929	0.110	0.199	
≥2	131/418 (31.3%)	0.719	0.677	0.313	0.477	
≥3	449/992 (45.2%)	0.805	0.554	0.452	0.623	
≥4	88/157 (56.0%)	0.968	0.133	0.560	0.718	
≥5	45/77 (58.4%)	0.989	0.050	0.584	0.737	
≥6	7/11 (63.6%)	0.998	0.008	0.636	0.777	
≥7	2/2 (100.0%)	1.0	0.0019	1.0	1.0	
**External data**						
0	3/88 (3.4 %)	0.0	1.0	0.034	0.065	0.616 (0.516–0.719)
≥1	4/104 (3.8 %)	0.171	0.911	0.038	0.074	
≥2	10/134 (7.4 %)	0.372	0.794	0.074	0.138	
≥3	6/114 (5.2%)	0.621	0.500	0.052	0.100	
≥4	4/37 (10.8%)	0.839	0.323	0.108	0.195	
≥5	2/38 (5.2 %)	0.905	0.205	0.052	0.100	
≥6	2/10 (20.0 %)	0.977	0.147	0.200	0.333	
≥7	3/6 (50.0 %)	0.994	0.088	0.500	0.677	

[a]NCR ratio was calculated as the number of NCR divided by the total number of patients in each score.
Abbreviation: NCR, non-curative resection; AUROC, area under the receiver operating characteristics curve; CI, confidence interval.

Supplementary Table S4. Performance of the non-curative resection prediction model between the seven machine learning models

**Internal data**	**LR**	**SVM**	**KNN**	**NB**	**XGB**	**RF**	**MLP**
LR	-	0.940	0.216	0.854	0.181	0.257	0.674
SVM		-	0.335	0.844	0.375	0.325	0.771
KNN			-	0.314	0.708	0.921	0.361
NB				-	0.532	0.455	0.950
XGB					-	0.801	0.341
RF						-	0.370
**External data**	**LR**	**SVM**	**KNN**	**NB**	**XGB**	**RF**	**MLP**
LR	-	0.425	0.269	0.109	0.432	0.855	0.877
SVM		-	0.213	0.079	0.768	0.342	0.363
KNN			-	0.817	0.144	0.419	0.316
NB				-	0.053	0.196	0.096
XGB					-	0.475	0.344
RF						-	0.912

Note, the performance of the area under the receiver operating characteristics between the seven machine learning models was compared by the Delong test. Abbreviation: LR, logistic regression; SVM, support vector machine; KNN, k-nearest neighbors; NB, naive bayes; XGB, extreme gradient boosting; RF, random forest; MLP, multilayer perceptron.

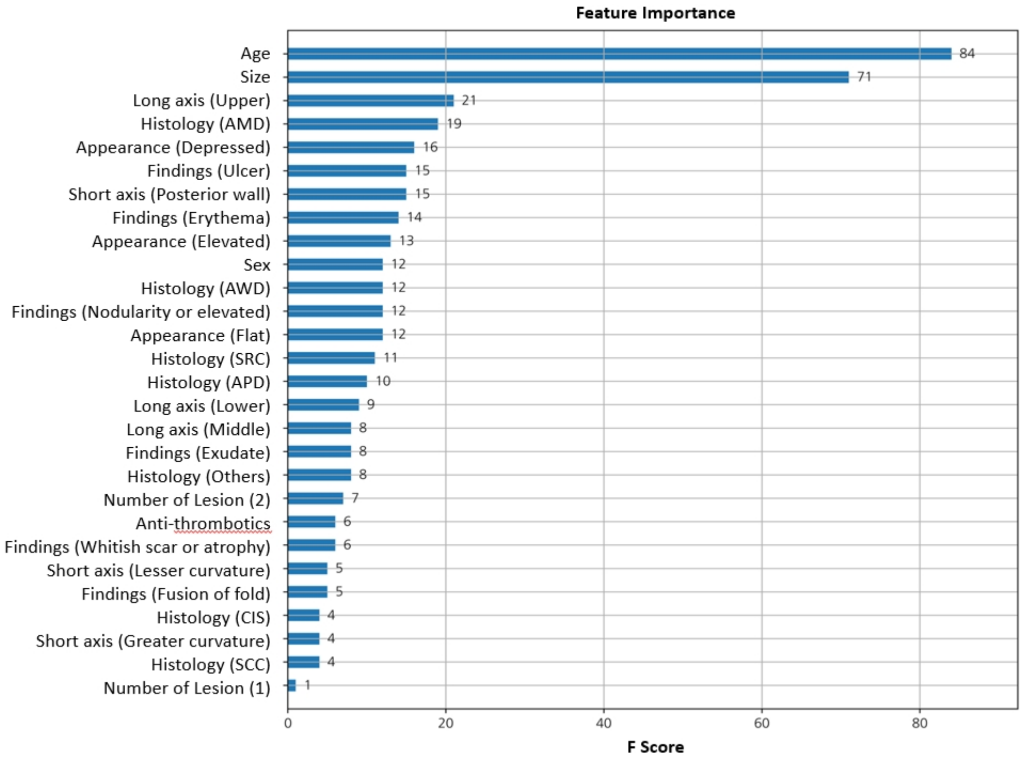

Supplementary Figure S1. Variables ordered by XGBoost feature importance and domain expertise Abbreviations: AMD, adenocarcinoma moderate-differentiated; AWD, adenocarcinoma well-differentiated; SRC, signet-ring cell; APD, adenocarcinoma poorly-differentiated; CIS, carcinoma in situ; SCC, squamous cell carcinoma.

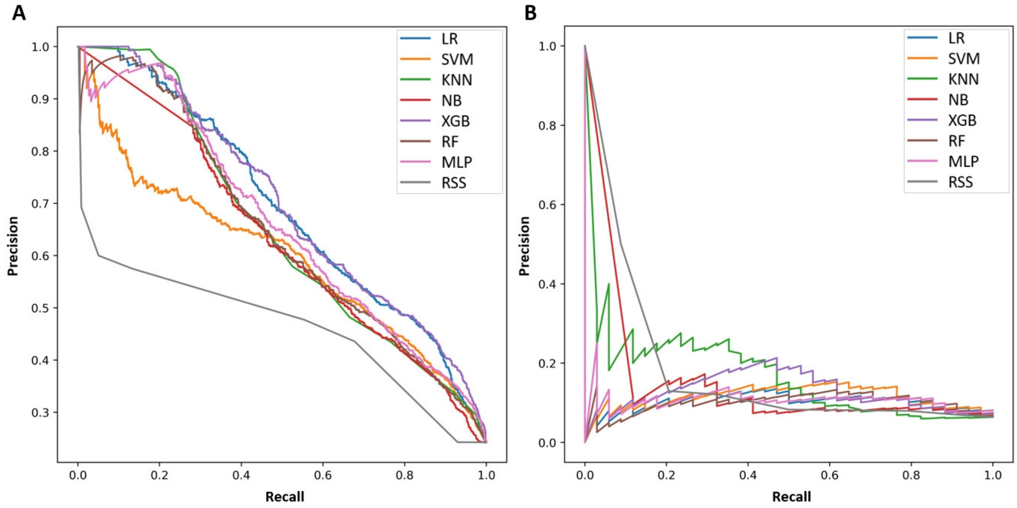

Supplementary Figure S2. The precision-recall curves for prediction of non-curative resection after endoscopic submucosal dissection (A) Internal data set, (B) External data set. Abbreviation: RSS, risk-scoring system; LR, logistic regression; SVM, support vector machine; KNN, k-nearest neighbors; NB, naive bayes; XGB, extreme gradient boosting; RF, random forest; MLP, multilayer perceptron.

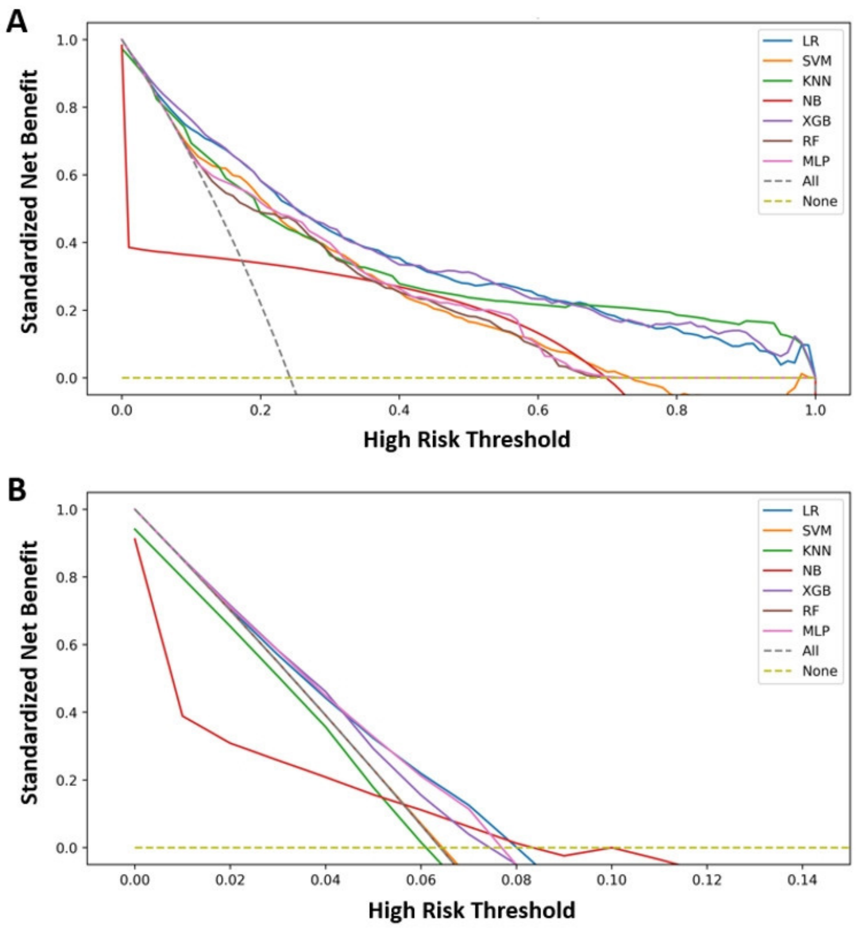

Supplementary Figure S3. Decision curve analysis of extreme gradient boosting and other models. (A) Internal data set, (B) External data set. Abbreviation: LR, logistic regression; SVM, support vector machine; KNN, k-nearest neighbors; NB, naive bayes; XGB, extreme gradient boosting; RF, random forest; MLP, multilayer perceptron.
